# Mobile phone addiction and insomnia among college students in China during the COVID-19 pandemic: a moderated mediation model

**DOI:** 10.3389/fpubh.2024.1338526

**Published:** 2024-03-11

**Authors:** Jinfu Wang, Xue Xu, Lijun Zuo, Haiyun Wang, Guan Yang

**Affiliations:** ^1^School of Physical Education, South China University of Technology, Guangzhou, China; ^2^School of Finance and Economy Guangdong Engineering Polytechnic, Guangzhou, China; ^3^School of Physical Education, Guangzhou College of Commerce, Guangzhou, China

**Keywords:** mobile phone addiction, insomnia, social anxiety, physical activity, moderated mediation model, college students, the COVID-19 pandemic

## Abstract

**Background:**

Nowadays, it is widely acknowledged that mobile phone addiction is a risky factor for insomnia symptoms, but to date, people know little about the underlying relationship between them among undergraduates during the COVID-19 pandemic. The purpose of the present study was to examine the potential association between mobile phone addiction and insomnia, as well as the mediating role of social anxiety and the moderating role of physical activity.

**Methods:**

Using the Mobile Phone Addiction Tendency Scale, Social Phobia Inventory, Physical Activity Rating Scale and Insomnia Severity Index, 301 eligible college students in China were investigated. For data analysis, descriptive analysis, correlation analysis, moderating effect test, moderating effect test were carried out in turn.

**Results:**

The findings revealed a favorable correlation between mobile phone addiction, social anxiety and insomnia, as well as between social anxiety and insomnia. But physical activity was negatively correlated with social anxiety and mobile phone addiction, and social anxiety partially mediated the relationship between mobile phone addiction and insomnia. Additionally, physical activity played a significant moderating effect between mobile phone addiction and social anxiety.

**Conclusion:**

This study advances the knowledge of how mobile phone addiction raises the likelihood of experiencing insomnia symptoms, and also implies that upping physical activity level could lessen the harmful impacts from mobile phone addiction.

## Introduction

Except for posing a serious threat to individual lives and respiratory function, the outbreak of novel coronavirus pneumonia (COVID-19) in late December 2019, has been also strongly linked to psychological and behavioral issues, such as depression, anxiety, and suicidal thoughts (1–3). Given that, the Chinese government implemented emergent measures to stop the spread of the COVID-19 pandemic due to the acute and contagious nature of this outbreak, including early isolation of confirmed or suspected cases, closure of schools and public transportation (4). While these emergent public health measures are highly necessary for reducing interpersonal transmission of COVID-19, there are still some possible reasons for concern as they may further lead to social isolation and other negative healthy outcomes (5). Researchers believe that there is a large number of potentials for using social media (e.g., mobile phones) during an epidemic lockdown, and that digital technology could be an effective way to alleviate public health needs and also provide social support (6), since it may be used online to communicate, access, learn, and receive the most lately information about the outbreak instead of face-to-face conversation (7).

Despite these latent benefits, it should be also kept in mind that excessive mobile phone use could produce lots of undesirable effects, especially the mobile phone addiction. As well all know, insomnia symptoms have been regarded as one of the main adverse outcomes generated by mobile phone addiction, with a series of cross-sectional studies showing that insomnia would be largely deteriorated with the escalation of mobile phone usage frequency (8–10). The longitudinal study has also demonstrated that problematic mobile phone use at baseline was a risky factor for adverse effects on students’ sleep and mental health, such as insomnia and depression during an 8 months follow-up survey (11).

However, at present, the key issue cannot be ignored by us would be that the underlying association between mobile phone addiction and insomnia among college students in the context of this emerging coronary pneumonia pandemic is poorly understood, because prior studies have mostly focused on the impact of mobile phone addiction on adolescents’ mental health. What’s worse, up till now, the probable mechanism in the relationship between mobile phone addiction and insomnia are still unknown for us. Given that, the current study attempts to built a moderated mediation model to evaluate the mediating role of social anxiety between mobile phone addiction and insomnia, and the moderating role of physical activity between mobile phone addiction and social anxiety among Chinese college students, so as to provide objective and persuasive answers for these concerns.

### The mediating role of social anxiety

Social anxiety refers to strong feelings of worry, unease, or fear in response to certain interpersonal situations (12), and numerous research has documented that social anxiety predicts depression and perceived stress in a positive way (13, 14). In turn, depression and stress perception would cause serious insomnia symptoms (15, 16). In addition, a study conducted by Buckner et al. found that social anxiety was significantly and unfavorably correlated with scores on measures of sleep-related dysfunction in American undergraduate students (17).

It can be obviously seen that social anxiety could be obviously influenced by mobile phone addiction. According to Clark and Wells’ cognitive model of social anxiety, persons with poor cognition and low self-esteem will be more prone to produce social anxiety (18). However, it has been discovered that mobile phone addiction may be strongly linked to cognitive dysfunction (19), and long-term and continuous exposure to electromagnetic radiation could also lower individuals’ working memory capacity and impair their attentional control, which in turn raises the risk of cognitive impairment (20). These findings suggest that using mobile phones excessively may not only result in cognitive impairment, but also evidently increase social anxiety. Therefore, based on the theoretical and empirical findings mentioned above, it is plausible to assume that mobile phone addiction could affect social anxiety. Given that, mobile phone addiction may lead to social anxiety, which then produces insomnia symptoms. Thus, the present study reasonably assumes that (H1): social anxiety possibly mediates the relationship between mobile phone addiction and insomnia.

### The moderating effect of physical activity

Actually, not all college students may be similarly impacted by mobile phone addiction, despite the fact that it could easily cause social anxiety and insomnia. However, it is also crucial to examine that whether other factors acting as a moderator influence the relationship between mobile phone addiction and insomnia which is mediated by the effect of social anxiety. Given that, the current study investigated whether a person’s amount of physical activity could affect the direct impact of mobile phone addiction on social anxiety. No doubt, physical activity has been widely defined as a variety of physical exercise forms that are planned and structured by the individuals, contracted by skeletal muscles, and usually caused manifest energy loss, with a strong subjective and directional nature (21). The previous work has shown that physical activity could improve levels of self-esteem associated with fear of negative evaluation (a core feature of social anxiety) (22, 23). Furthermore, social anxiety can be effectively improved by participating in an 8-week moderate-intensity aerobic exercise program, and this positive and desirable effect is more likely to happen to those guys with high levels of social anxiety (24). And several studies have also demonstrated that actively engaging in physical activity might lessen the detrimental consequences from addictive behaviors on people’s physical and mental health. For instance, Tao et al. (25) discovered that physical activity could mediate the association between mobile phone addiction and sadness, with the association being higher for individuals with lower physical activity.

Therefore, in accordance with those persuasive and reliable evidence, it can be naturally inferred that physical activity would potentially contribute to the relationship between mobile phone addiction and its negative effects by mitigating risk (e.g., social anxiety). That is, physical activity could influence the link between mobile phone addiction and social anxiety in a large degree. Given that, the present study assumes that (H2): physical activity may play a moderating role in the relationship between mobile phone addiction and social anxiety.

### The moderated mediation model

**Figure 1:**
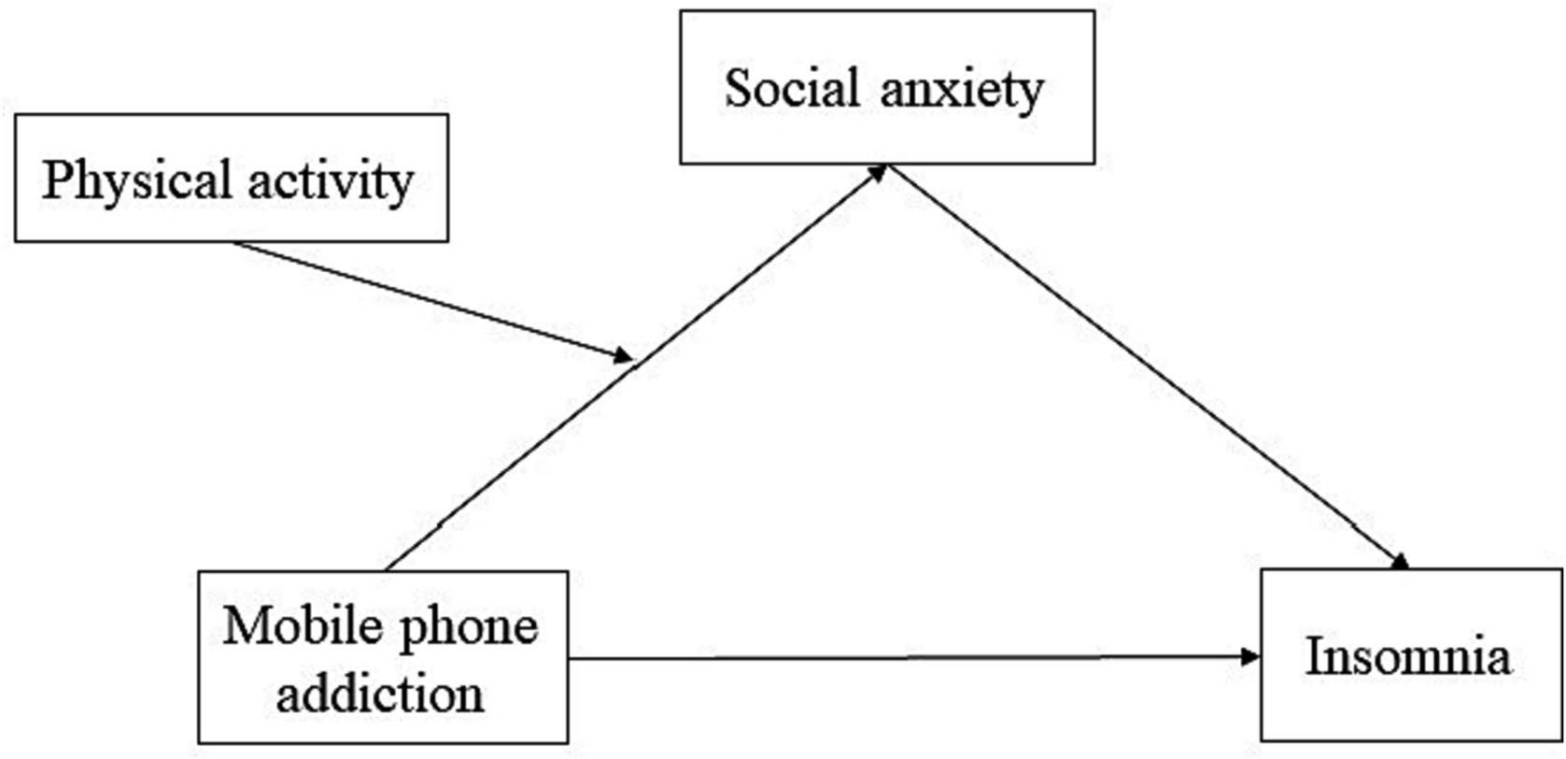
The hypothesized model between mobile phone addiction and insomnia.

In summary, in the light of those analyses mentioned above, the purpose of this study was to testify the latent mechanism that underlies the correlation between mobile phone addiction and insomnia among college students. In other words, the current work developed a moderated mediation model to address two issues: (a) social anxiety could or not mediate the association between mobile phone addiction and insomnia, and (b) whether physical activity could moderate the relationship between mobile phone addiction and social anxiety. The below moderated mediation model in this study (Figure 1) can be used to properly explain how mobile phone addiction affects insomnia, and how to moderate the relationship between mobile phone addiction and social anxiety by physical activity.

## Methods

### Procedures and participants

Given the emergence of a new crown epidemic at that hard time and the drawbacks from interpersonal social interaction, this cross-sectional study with an online questionnaire was finally completed by us in the middle of January 2022; and then, a convenient sampling was conducted in Shandong University, a comprehensive university affiliated with the Ministry of the Education in China, which has 55 colleges covering thirteen broad disciplines, including philosophy, economics, law, education, literature, history, science, engineering, agriculture, medicine, management, military science, and art. At last, 301 eligible college students with the mean age of 20.47 (SD = 4.99) were luckily chosen and then successfully filled out their online questionnaires. Before completing that, they were also informed and highlighted that this online survey was totally anonymous and voluntary, and they need to sign the informed consent first. To confirm the accuracy and validity of the data, the researchers seriously and carefully checked each questionnaire’s completed data after it had been filled out for internal logic and consistency.

The present work involving human participants was carried out in accordance with the 1964 Declaration of Helsinki and its later amendments or comparable ethical standards, and also reviewed and approved by the research ethics committee at South China University of Technology. And all participants had provided their online informed consent before formally engaging in this survey.

## Measures

### Study design

This cross-sectional study aimed to explore the relationships among mobile phone addiction, social anxiety, insomnia, and physical activity among college students during the COVID-19 pandemic.

### Mobile phone addiction

The degree of mobile phone addiction among college students was assessed using the Chinese version of the Mobile Phone Addiction Tendency Scale (MPATS), originally developed by Xiong et al. (26). The scale has 16 items in four dimensions. College students rated each item on a 5-point-Likert scale, ranging from 1 (very non-compliant) to 5 (very compliant). There were no reverse scoring items, and the total score was determined by adding up the scores from the 16 items. Higher score would indicate a higher likelihood of mobile phone addiction. This measure has been widely used in Chinese university populations and has shown good reliability and validity (27). For the current study, the test showed good reliability (Cronbach’s *α* = 0.884).

### Social anxiety

Social anxiety among college students was assessed using the Chinese version of the Social Phobia Inventory (SPIN) (28). The scale has 17 items in three aspects. Participants rated each item on a 5-point-Likert scale ranging from 0 (not at all) to 4 (extremely), with higher score indicating more severe social anxiety symptoms. This measure has been widely used in Chinese university populations and has shown good reliability and validity (29). The Cronbach’s alpha coefficient of this scale displayed good reliability in the current study (Cronbach’s *α* = 0.934).

### Physical activity

Physical activity levels were assessed using the Physical Activity Rating Scale (PARS-3) (30). This scale has 3 items (intensity, time, and frequency), and the amount of physical activity was calculated by the following formula: “exercise intensity × (exercise duration − 1) × exercise frequency.” The total scores of physical activity amount were from 0 to 100. This measure has been widely used in the Chinese university populations and has shown good reliability and validity (31, 32). The internal consistency of the PARS-3 in this study was generally satisfactory, with the Cronbach’s alpha coefficient of 0.702.

### Insomnia

Insomnia symptoms were assessed by the Insomnia Severity Index (ISI) (33), a scale that has been validated in Chinese youth populations and totally meets psychometric requirements (34). Each item of the ISI was rated on a 5-point-Likert scale (0 = none, 4 = very severe), and responses to the seven items were summed to obtain the total score of ISI, with higher scores indicating higher levels of insomnia symptoms. In the present study, the Cronbach’s alpha coefficient for ISI was 0.715.

### Demographic variables

Except the four main variables mentioned above, the current study also investigated several demographic indicators about the participants, including age, gender, grade, place of residence, self-evaluation of academic performance, and daily sleep time by using a standard form.

### Statistical analysis

All statistical analyses were performed using SPSS 26.0 software. Continuous variables were presented as mean and standard deviation (M ± SD), and categorical variables displayed as frequency (*n*) and percentage (%). If the continuous variable does not conform to the normal distribution, the normality transformation would be adopted by processing the root number. In this study, we first analyzed the general characteristics of participants. Secondly, preliminary analyses were performed to explore the potential correlation between mobile phone addiction, social anxiety, physical activity, and insomnia. The third step was to examine the mediating role of social anxiety between mobile phone addiction and insomnia through the SPSS macro-PROCESS model 4, and likewise, SPSS macro-PROCESS model 7 was utilized to assess the moderating role of physical activity between mobile phone addiction and social anxiety. In this work, 5,000 bootstrapped samples were drawn from the data, and 95% bootstrap upper and lower confidence intervals (CI) were also calculated. Before testing the mediating and moderating effect, the four main variables were all centralized in advance, and as the control variable, gender and grade was all put into the measurement model. The significance level in the present study was set at alpha = 0.05.

## Results

### Common method bias

Due to the use of self-report scales for all variables in this study, there is a potential for common method bias. To examine whether such bias exists, the data underwent a Harman’s single-factor test for homogeneity of variances. The first factor, without rotation, contributed 34.98% to the total loading, which is below the 40% threshold. This suggests that there is no significant common method bias in this study.

### Demographic variables

As shown in Table 1, the sample for this study consisted of 301 college students, which comprises 174 male (57.8%) students and 127 female (42.2%) students. Of them, 73 students (24.3%) were from rural areas and 228 students (75.7%) from urban areas, and they were also distributed in five grades, namely freshmen (12.6%), sophomore (22.9%), junior (29.9%), senior (28.2%), and postgraduate (6.4%). In terms of academic performance, 39 students (13.0%) considered their academic performance poor, 180 students (57.8%) considered their academic performance average, and 82 students (27.2%) considered their academic performance good. About daily sleep time, 25 students (8.3%) were less than 6 h per day, 239 students (79.4%) were from 6 to 8 h per day, and 37 students (12.3%) were more than 8 h per day.

**Table 1:** General characteristics of participants.

Variables	Categories	*n*	%
Gender	Male	174	57.8
	Female	127	42.2
Grade	Freshman	38	12.6
	Sophomore	69	22.9
	Junior	90	29.9
	Senior	85	28.2
	Postgraduate	19	6.4
Place of residence	Rural	73	24.3
	Urban	228	75.7
Self-evaluation of academic performance	Poor	39	13.0
	Average	180	57.8
	Good	82	27.2
Daily sleep time	≤6 h	25	8.3
	6–8 h	239	79.4
	≥8 h	37	12.3

### Preliminary analyses

Descriptive statistics and correlation analysis for mobile phone addiction, social anxiety, physical activity, and insomnia are shown in Table 2. Due to the variable of physical activity was not satisfactory with normal distribution, so the corresponding normality transformation had been firstly performed on it. The results showed that mobile phone addiction was positively correlated with social anxiety (*p* < 0.01) and insomnia (*p* < 0.01), and social anxiety was positively correlated with insomnia (*p* < 0.01). Physical activity was negatively correlated with social anxiety (*p* < 0.05) and mobile phone addiction (*p* < 0.05), and there was no statistically significance between physical activity and insomnia (*p* > 0.05).

**Table 2:** Descriptive statistics and correlation analysis for main variables.

Variables	M	SD	1	2	3	4
1. Mobile phone addiction	48.15	10.86	–			
2. Social anxiety	40.07	12.89	0.32^**^	–		
3. Physical activity	3.47	1.40	−0.12^*^	−0.12^*^	–	
4. Insomnia	18.91	5.32	0.25^**^	0.21^**^	−0.03	–

M, mean; SD, standard deviation. ^*^p < 0.05 and ^**^p < 0.01.

### Mediating effect test

As shown in Table 3, after controlling for gender and grade, mobile phone addiction was positively correlated with social anxiety (*β* = 0.32, *p* < 0.001), and social anxiety was also positively associated with insomnia (*β* = 0.14, *p* < 0.01). The total effect between mobile phone addiction and insomnia was significant (*β* = 0.25, *p* < 0.001), and meanwhile, the indirect effect between them was also remarkably significant (indirect effect = 0.03, SE = 0.01, 95% Boot CI = 0.01–0.06), suggesting that social anxiety could partially mediate the correlation between mobile phone addiction and insomnia. Thus, it can be clearly seen that social anxiety played a mediating role in the relationship of mobile phone addiction on insomnia.

**Table 3:** Testing the mediating effect of social anxiety between mobile phone addiction and insomnia.

Outcome variable	Predictors	*R* ^2^	*F*	*β*	LLCI	ULCI	*t*
Insomnia	Gender	0.07	7.71^***^	−0.06	−0.21	0.06	−1.11
	Grade			−0.05	−0.08	0.02	−1.01
	Mobile phone addiction			0.25	0.11	0.29	4.59^***^
Social Anxiety	Gender	0.12	13.88^***^	−0.10	−0.44	−0.00	−2.00
	Grade			0.03	−0.05	0.12	0.72
	Mobile phone addiction			0.32	0.28	0.55	5.98^***^
Insomnia	Gender	0.09	7.46^***^	−0.04	0.04	−0.08	−0.82
	Grade			−0.06	−0.08	0.02	−1.12
	Mobile phone addiction			0.20	0.07	0.25	3.55^***^
	Social Anxiety			0.14	0.01	0.16	2.51^**^

*β*, standardized regression coefficient; LLCI, lower level CI; ULCI, upper level CI. ^**^p < 0.01 and ^***^p < 0.001.

### Moderating effect test

**Figure 2:**
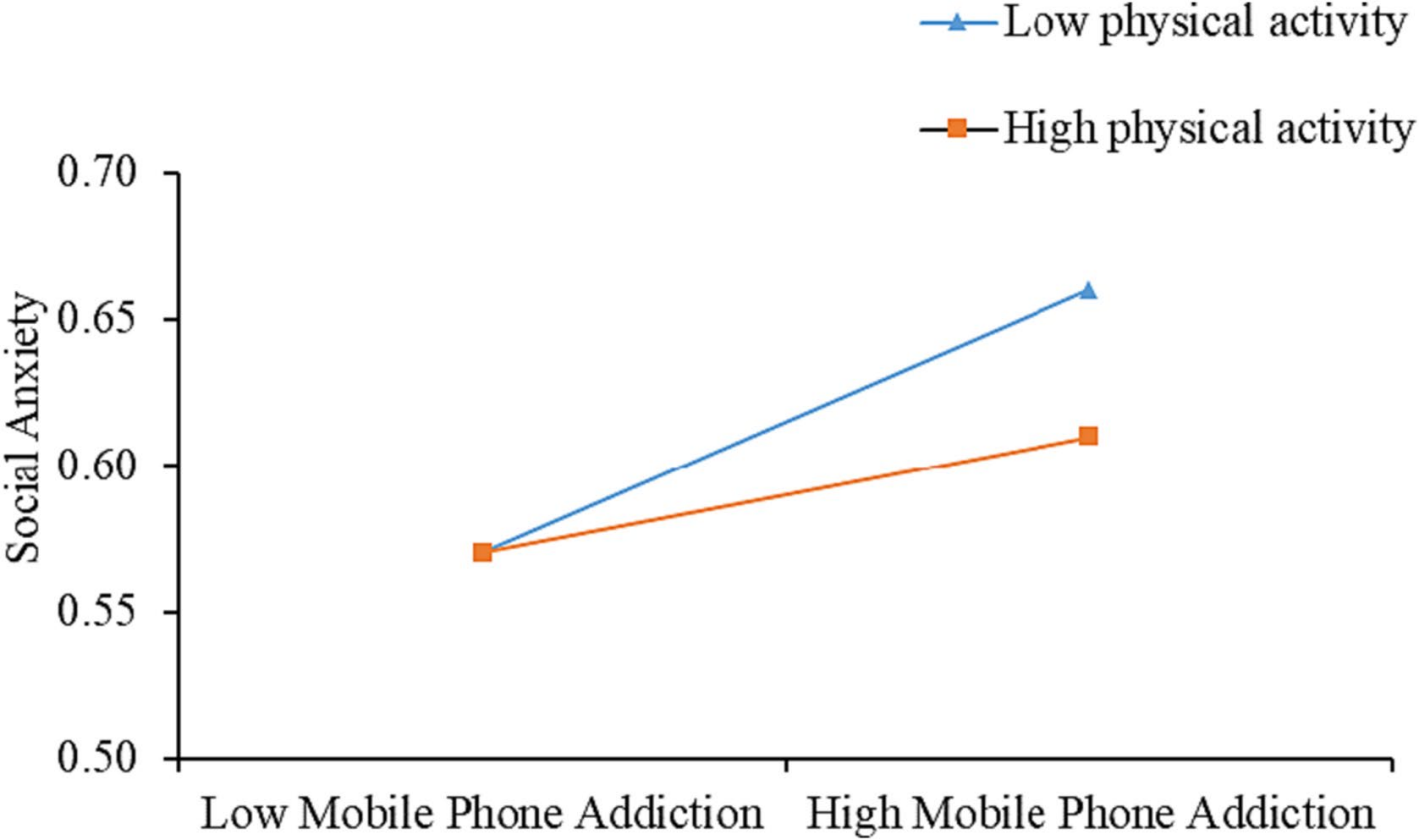
Physical activity as a moderator between mobile phone addiction and social anxiety.

As shown in Table 4, a significant direct effect of mobile phone addiction on social anxiety was observed after controlling for gender and grade (*β* = 0.41, *p* < 0.001), and this effect was obviously moderated by physical activity (*β* = −0.05, *p* < 0.05). The results of the conditional direct effects analyses were depicted in Figure 2, which shows the relationship between mobile phone addiction and social anxiety at low and high levels of physical activity, namely the mean below 1SD or above 1SD, respectively. Likewise, a simple slope test also displayed that physical activity had a moderating effect on the impact of mobile phone addiction on social anxiety, and compared to the high physical activity, that was evidently stronger for college students with low levels of physical activity.

**Table 4:** Testing the moderating effect of physical activity between mobile phone addiction and social anxiety.

Outcome variable	Predictors	*R* ^2^	*F*	*β*	LLCI	ULCI	*t*
Social anxiety	Gender	0.15	10.51^***^	−0.28	−0.05	−0.06	−2.55^*^
	Grade			0.01	−0.07	0.10	0.04
	Mobile phone addiction			0.41	0.27	0.55	5.94^***^
	Physical activity			−0.05	−0.09	−0.00	−2.21^*^
	Mobile phone addiction × physical activity			−0.05	−0.10	−0.01	−2.38^*^
				*β*	Boot SE	Boot LLCI	Boot ULCI
Conditional direct effect analysis at three kinds of moderator values = M ± 1SD							
M − 1SD (1.07)				0.55^***^	0.09	0.36	0.73
M (3.47)				0.41^***^	0.07	0.27	0.55
M + 1SD (5.87)				0.27^***^	0.08	0.10	0.44

M, mean; SD, standard deviation; *β*, standardized regression coefficient; LLCI, lower level CI; ULCI, upper level CI. ^*^p < 0.05 and ^***^p < 0.001.

## Discussion

The present study focused on the potential correlation between mobile phone addiction and insomnia, and the mediating effect of social anxiety and the moderating effect of physical activity possibly between them among college students in China. The final results demonstrated that mobile phone addiction was positively associated with insomnia, which is consistent with preceding research (9, 10). Additionally, social anxiety was also positively correlated with mobile phone addiction and insomnia, respectively, and meanwhile, social anxiety could mediate the relationship between them. Therefore, the hypothesis 1 (H1) of the current work has been fairly proved. This finding can be explained via the cognitive-behavioral model of social anxiety, which points out that individuals’ early traumatic experiences would result in the development of unfavorable irrational beliefs and self-perceptions, as well as additional unfavorable cognitive processing biases, which might easily lead to emotional (such as anxiety) and behavioral (such as daytime sleepiness) issues (35). The cognitive model of insomnia maintenance also proposes that anxiety situations induce people to selectively pay attention to and monitor internal and external threat cues closely correlated with sleep, which would largely exacerbate the insomnia symptoms (36). But it is worth noting that social anxiety could only partially account for the relationship between mobile phone addiction and insomnia; thus other key latent indicators, such as interpersonal distress, should also considered when examining the mediation role between them afterwards.

Apart from the overall analyses, no doubt, the two essential processes of this mediating effect between mobile phone addiction and insomnia have been also equally noteworthy. On the one hand, in the first stage of this mediation, it can be easily found that college students with higher degree of mobile phone addiction also had more awfully social anxiety, which is in line with previous research findings (37, 38) and suggests that mobile phone addiction is an important risky factor for social anxiety. The previous research has also indicated that those severely addicted to mobile phones had poor interpersonal relationship and social communication skills in general, which might worsen their social anxiety and then cause an obvious decline in their interpersonal communicating skills in the real world, as well as eventually resulting in social anxiety (39). On the other hand, for the second stage of the mediation, it was not hard to find that college students with higher level of social anxiety would be more prone to produce insomnia, which is consistent with prior studies (40, 41). One possible explanation might be that higher level of social anxiety can lead to emotional distress and impairments in various functional domains (17), including depression and negative peer relationships (35, 42). Likewise, the previous research has also shown that depression and poor interpersonal relationships are important factors to predict insomnia (42, 43). Consequently, individuals with higher level of social anxiety may have significant symptoms of insomnia. Undoubtedly, all these evidence from the previous work persuasively support the mediating role of social anxiety played in the relationship between mobile phone addiction and insomnia.

At the same time, this research has also found that physical activity was inversely correlated with mobile phone addiction and social anxiety, respectively, and meanwhile, physical activity could significantly moderate the relation between them. Thus, the hypothesis 2 (H2) of the present study has been fairly proved, too. According to this finding, it can be properly inferred that mobile phone addiction does not always result in the same degree of behavioral issues, such as social anxiety, and that some healthy habits or lifestyles including the active participation with physical activity, may help persons to effectively lessen the negative impacts from mobile phone addiction. The following factors, in particular, could be utilized to interpret how does physical activity moderate the association between mobile phone addiction and social anxiety.

Firstly, engaging in physical activity could help people manage their negative emotions, such as loneliness and stress, and also enhance their psychological capital in terms of their mental health (44, 45). However, emotional issues, such as the symptoms of loneliness and stress, might contribute to generate recurrent social anxiety (46, 47). That is, compared to addictive individuals with low and even no physical activity level, those frequently engaging in physical activity and acquiring the higher level of physical activity, would be more likely to possess desirable mental health and great psychological capital, and as a result, the harmful impacts from mobile phone addiction might be considerably decreased for them. Secondly, actively participating in physical activity helps the person build “intrinsic assets” and “extrinsic resources,” such as self-efficacy, self-esteem, parental support, and the good peer relationships (48, 49); and actually, these advantages have all been demonstrated to form defense mechanisms against mobile phone addiction (50, 51). According to the protective model of resilience (52), people can lessen or minimize the impact of risky exposure on adverse outcomes by increasing their assets (e.g., self-esteem) and resources (e.g., parental support); accurately speaking, when individuals are exposed to risky settings or conditions, they may still have the ability to show positive performance against these adverse situations. It is no denying that physical activity would be a feasible and reliable means to help human beings obtain this so-called resilience. Therefore, as for those addicted to mobile phones, acquiring the higher level of physical activity may be the most likely to prevent, resist or avoid the passive effects from mobile phone addiction.

### Limitations and future directions

It is crucial to underscore several limitations inherent in the present study, which need to be taken into account in coming days. Firstly, the utilization of a cross-sectional design employed in our study precludes the establishment of causal relationships. Therefore, to enhance the robustness of our findings, a longitudinal design and experimental protocols could be implemented. Secondly, the data for this study had to be collected online due to social restrictions during the COVID-19 period, leading to the exclusion of college students without internet access. Therefore, the experimental study could be conducted to objectively acquire relevant data in coming days, such as physical activity, and mobile phone addiction (53). Thirdly, relying on self-reported data introduces the potential for recall bias. To mitigate this limitation, future research could gain advantages from incorporating objective and effective instruments. For example, utilizing tools such as 3D-sensor pedometers, ActiGraph GT3X + accelerometers, and artificial sport bracelets could improve the accuracy and reliability of data, especially in the measurement of physical activity. Fourthly, the exclusive concentration on college students during a specific period of the new coronavirus pandemic raises concerns regarding the generalizability of our results to other populations or normal conditions. This necessitates additional external validation to confirm the external validity of the current study. Additionally, employing convenient sampling led to a moderate sample size. To enhance the methodological rigor of the study, future research should consider adopting probability sampling methods and larger sample sizes. Lastly, while our study centered on social anxiety, future researchers may explore analogous latent variables related to mobile phone addiction and insomnia as potential mediators. This approach would contribute to a more thorough understanding of the underlying mechanisms between these variables.

### Implications

Regardless of those drawbacks mentioned above, the current study has also made some original contributions and a number of significant practicable implications. To our knowledge, this work is the first one to examine how social anxiety mediates the relationship of mobile phone addiction and insomnia, and the moderating effect of physical activity between mobile phone addiction and social anxiety, which has largely expanded the previous research. According to the analyses regarding the moderated mediation model, the potential mechanism, namely how mobile phone addiction affects insomnia among college students, has been perfectly disclosed and clearly told us that how does mobile phone addiction cause insomnia symptoms, and the feasible way to lessen the connection between mobile phone addiction and social anxiety. More important, based on these important findings, several essential practicable meanings of this study can be naturally concluded. First and foremost, aiming to make college students aware of the detrimental effects of mobile phone addiction on insomnia symptoms, parents and teachers should emphasize the latent correlation between mobile phone addiction and insomnia. Furthermore, given that social anxiety plays a pivotal mediating role between mobile phone addiction and insomnia, parents and teachers should employ practical approaches to assist students in acquiring effective strategies for managing their social anxiety in daily life. This may include engaging in communication dialogues, providing emotional support, and implementing dynamic cognitive-behavioral psychotherapy (54). Lastly, considering that physical activity has the potential to alleviate the adverse impact of mobile phone addiction on social anxiety, and higher levels of physical activity may be more beneficial in moderating social anxiety. Hence, college students should proactively engage in various exercise programs on campus. Simultaneously, they should strive to enhance their physical activity levels by increasing exercise intensity or frequency. Additionally, consistent with prior research, it is advisable to integrate additional motivational learning tasks into routine physical education content. This approach can effectively boost college students’ motivation to embrace healthy behaviors (55), thereby enhancing their physical activity levels and alleviating the negative repercussions of mobile phone addiction.

## Conclusion

The current study reveals the mechanism of association between smartphone addiction and insomnia. Our research found that smartphone addiction among college students is significantly positively correlated with social anxiety and insomnia, and significantly negatively correlated with physical activity. Social anxiety plays a mediating role to some extent in the relationship between smartphone addiction and insomnia. Furthermore, physical exercise plays a significant moderating role between smartphone addiction, social anxiety, and insomnia. In summary, our research results indicate that increasing physical activity and reducing social anxiety can improve insomnia symptoms among college students. To prevent future problematic behaviors among college students, it is necessary to adopt proactive health behaviors, such as engaging in physical activity, to counteract or avoid subsequent passive impacts.

## Data availability statement

The original contributions presented in the study are included in the article/supplementary material, further inquiries can be directed to the corresponding authors.

## Ethics statement

The studies involving humans were approved by South China University of Technology. The studies were conducted in accordance with the local legislation and institutional requirements. The participants provided their written informed consent to participate in this study.

## Author contributions

JW: Conceptualization, Data curation, Formal analysis, Investigation, Methodology, Software, Supervision, Writing – original draft, Writing – review & editing. XX: Conceptualization, Methodology, Writing – review & editing. LZ: Investigation, Writing – review & editing. HW: Conceptualization, Data curation, Writing – review & editing. GY: Conceptualization, Methodology, Supervision, Writing – review & editing.

## References

[1] Huckins JF daSilva AW Wang W Hedlund E Rogers C Nepal SK. Mental health and behavior of college students during the early phases of the COVID-19 pandemic: longitudinal smartphone and ecological momentary assessment study. J Med Internet Res. (2020) 22:e20185. doi: 10.2196/20185, PMID: 32519963 PMC7301687

[2] Li G Conti AA Qiu C Tang W. Adolescent mobile phone addiction during the COVID-19 pandemic predicts subsequent suicide risk: a two-wave longitudinal study. BMC Public Health. (2022) 22:1537. doi: 10.1186/s12889-022-13931-1, PMID: 35962376 PMC9372972

[3] Zhang Y Zhang H Ma X Di Q. Mental health problems during the COVID-19 pandemics and the mitigation effects of exercise: a longitudinal study of college students in China. Int J Environ Res Public Health. (2020) 17:3277. doi: 10.3390/ijerph17103722, PMID: 32466163 PMC7277113

[4] Liu Y Wang L Chen L Zhang X Bao L Shi Y. Mental health status of Paediatric medical Workers in China during the COVID-19 outbreak. Front Psych. (2020) 11:702. doi: 10.3389/fpsyt.2020.00702, PMID: 32792998 PMC7385285

[5] Liang SW Liu LL Peng XD Chen JB Huang AD Wang XY. Prevalence and associated factors of suicidal ideation among college students during the COVID-19 pandemic in China: a 3-wave repeated survey. BMC Psychiatry. (2022) 22:336. doi: 10.1186/s12888-022-03968-2, PMID: 35570282 PMC9107580

[6] Ammar A Bouaziz B Trabelsi K Glenn JM Zmijewski P Müller P. Applying digital technology to promote active and healthy confinement lifestyle during pandemics in the elderly. Biol Sport. (2021) 38:391–6. doi: 10.5114/biolsport.2021.100149, PMID: 34475622 PMC8329971

[7] Iyengar K Upadhyaya GK Vaishya R Jain V. COVID-19 and applications of smartphone technology in the current pandemic. Diabetes Metab Syndr Clin Res Rev. (2020) 14:733–7. doi: 10.1016/j.dsx.2020.05.033, PMID: 32497963 PMC7248636

[8] Al Battashi N Al Omari O Sawalha M Al Maktoumi S Alsuleitini A Al QM. The relationship between smartphone use, insomnia, stress, and anxiety among university students: a cross-sectional study. Clin Nurs Res. (2021) 30:734–40. doi: 10.1177/1054773820983161 33375850

[9] Honglv X Jian T Jiaxing Y Yunpeng S Chuanzhi X Mengdie H. Mobile phone use addiction, insomnia, and depressive symptoms in adolescents from ethnic minority areas in China: a latent variable mediation model. J Affect Disord. (2023) 320:381–9. doi: 10.1016/j.jad.2022.09.156, PMID: 36206877

[10] Zhang C Hao J Liu Y Cui J Yu H. Associations between online learning, smartphone addiction problems, and psychological symptoms in Chinese college students after the COVID-19 pandemic. Front Public Health. (2022) 10:881074. doi: 10.3389/fpubh.2022.881074, PMID: 35602144 PMC9114473

[11] Liu S Wing YK Hao Y Li W Zhang J Zhang B. The associations of long-time mobile phone use with sleep disturbances and mental distress in technical college students: a prospective cohort study. Sleep. (2019) 42:42. doi: 10.1093/sleep/zsy213, PMID: 30395300

[12] Guo XW. A study on the causes of social anxiety among college students. Exp Psychol. (2000) 1:55–8.

[13] Ingram RE Ramel W Chavira D Scher C. Social anxiety and depression, Crozier W. R. Alden L. E. (Eds.), International handbook of social anxiety. Chichester, United Kingdom, (2001) 357–280

[14] Yamaguchi A Kim M-S Akutsu S Oshio A. Effects of anger regulation and social anxiety on perceived stress. Health Psychol Open. (2015) 2:2055102915601583. doi: 10.1177/2055102915601583, PMID: 28070369 PMC5193268

[15] Hua Z Ma D Xia X. Emotional dysregulation and time structure mediate the link between perceived stress and insomnia among unemployed young people in China: a cross-sectional study. Int J Environ Res Public Health. (2022) 19:11883. doi: 10.3390/ijerph191911883, PMID: 36231183 PMC9564838

[16] Kabir A Brinsworth J. Prevalence of depression, anxiety, stress, and insomnia in Iranian gay men during the COVID-19 pandemic. J Homosex. (2022) 71:632–44. doi: 10.1080/00918369.2022.2130022 36269157

[17] Buckner JD Bernert RA Cromer KR Joiner TE Schmidt NB. Social anxiety and insomnia: the mediating role of depressive symptoms. Depress Anxiety. (2008) 25:124–30. doi: 10.1002/da.20282 17340615

[18] Clark DM Wells A. A cognitive model of social phobia. New York: Guilford Press (1995).

[19] Hu Y Huang H Zhang YQ Zhou CY. The mediating effect of negative emotions between Mobile phone dependence and cognitive failure. Chin J Clin Psychol. (2017) 6:1088–92. doi: 10.16128/j.cnki.1005-3611.2017.06.020

[20] Hadlington LJ. Cognitive failures in daily life: exploring the link with internet addiction and problematic mobile phone use. Comput Hum Behav Rep. (2015) 51:75–81. doi: 10.1016/j.chb.2015.04.036

[21] Caspersen CJ Powell KE Christenson GM. Physical activity, exercise, and physical fitness: definitions and distinctions for health-related research. Public Health Rep. (1985) 100:126–31. 3920711 PMC1424733

[22] Bei Y Dan L Tong-Tong D Jun-Sheng L Xin-Yin C. Developmental trajectories and influencing factors of fear of negative evaluation in adolescence (revised). Psychol Sci. (2019) 42:62–7. doi: 10.16719/j.cnki.1671-6981.20190110

[23] Yìğìter K. The effects of participation in regular exercise on self-esteem and hopelessness of female university students. Soc Behav Pers. (2014) 42:1233–43. doi: 10.2224/sbp.2014.42.8.1233

[24] Jazaieri H Lee IA Goldin PR Gross JJ. Pre-treatment social anxiety severity moderates the impact of mindfulness-based stress reduction and aerobic exercise. Psychol Psychother Theory Res Pract. (2016) 89:229–34. doi: 10.1136/bjsports-2012-091287, PMID: 25684277 PMC4537407

[25] Tao S Wu X Yang Y Tao F. The moderating effect of physical activity in the relation between problematic mobile phone use and depression among university students. J Affect Disord. (2020) 273:167–72. doi: 10.1016/j.jad.2020.04.012, PMID: 32421598

[26] Xiong J Zhou ZK Chen W You ZQ Zhai ZY. Development of the mobile phone addiction tendency scale for college students. Chin Ment Health J. (2012) 26:222–5. doi: 10.3969/j.issn.1000-6729.2012.03.013

[27] Yang G Tan GX Li YX Liu HY Wang ST. Physical exercise decreases the Mobile phone dependence of university students in China: the mediating role of self-control. Int J Environ Res Public Health. (2019) 16:4098. doi: 10.3390/ijerph16214098, PMID: 31652978 PMC6862431

[28] Tsai CF Wang SJ Juang KD Fuh JL. Use of the Chinese (Taiwan) version of the social phobia inventory (SPIN) among early adolescents in rural areas: reliability and validity study. Int J Environ Res Public Health. (2009) 72:422–9. doi: 10.1016/S1726-4901(09)70399-5, PMID: 19686998

[29] Liu Z Li M Ren C Zhu G Zhao X. Relationship between physical activity, parental psychological control, basic psychological needs, anxiety, and mental health in Chinese engineering college students during the COVID-19 pandemic. Front Psychol. (2022) 13:802477. doi: 10.3389/fpsyg.2022.802477, PMID: 35350737 PMC8958037

[30] Liang DQ Liu SJ. The relationship between stress level and physical exercise for college students. Chin Ment Health J. (1994) 8:5–6.

[31] Yang G Li YX Liu SJ Liu CN Jia C Wang ST. Physical activity influences the mobile phone addiction among Chinese undergraduates: the moderating effect of exercise type. J Behav Addict. (2021) 10:799–810. doi: 10.1556/2006.2021.00059, PMID: 34546969 PMC8997213

[32] Ke YY Liu XX Xu X He BC Wang JF Zuo LJ. Self-esteem mediates the relationship between physical activity and smartphone addiction of Chinese college students: a cross-sectional study. Front Psychol. (2024) 14:1256743–51. doi: 10.3389/fpsyg.2023.1256743, PMID: 38250119 PMC10797096

[33] Bastien CH Vallières A Morin CM. Validation of the insomnia severity index as an outcome measure for insomnia research. Sleep Med. (2001) 2:297–307. doi: 10.1016/s1389-9457(00)00065-4 11438246

[34] Li EZ Li WX Xie ZT. Psychometric characteristics of insomnia severity index scale applied to students in business schools. J Neurosci Ment Health. (2019) 19:268–72. doi: 10.3760/cma.j.issn.1672-7088.2018.28.005

[35] Rapee RM Magson NR Forbes MK Richardson CE Johnco CJ Oar EL. Risk for social anxiety in early adolescence: longitudinal impact of pubertal development, appearance comparisons, and peer connections. Behav Res Ther. (2022) 154:104126. doi: 10.1016/j.brat.2022.104126, PMID: 35642989

[36] Harvey AG. A cognitive model of insomnia. Behav Res Ther. (2002) 40:869–93. doi: 10.1016/S0005-7967(01)00061-4 12186352

[37] Billieux J. Problematic use of the mobile phone: a literature review and a pathways model. Curr Pediatr Rev. (2012) 8:299–307. doi: 10.2174/157340012803520522

[38] Billieux J Maurage P Lopez-Fernandez O Kuss DJ Griffiths MD. Can disordered mobile phone use be considered a behavioral addiction? An update on current evidence and a comprehensive model for future research. Curr Addict Rep. (2015) 2:156–62. doi: 10.1007/s40429-015-0054-y

[39] Li ZB Wang TT Liang Y Wang MH. The relationship between mobile phone addiction and subjective well-being among college students: The mediating role of social anxiety. Stud Psychol Behav. (2017) 15:562–8.

[40] Attridge M. Internet-based cognitive-behavioral therapy for employees with anxiety, depression, social phobia, or insomnia: clinical and work outcomes. Sage Open Med. (2020) 10:215824402091439. doi: 10.1177/2158244020914398

[41] Malaeb D Salameh P Barbar S Awad E Hallit S. Problematic social media use and mental health (depression, anxiety, insomnia) among Lebanese adults: any mediating effect of stress? Perspect Psychiatr Care. (2020) 57:539–49. doi: 10.1111/ppc.12576, PMID: 32633428

[42] Raffray T Bond TL Pelissolo A. Correlates of insomnia in patients with social phobia: role of depression and anxiety. Psychiatry Res. (2011) 189:315–7. doi: 10.1016/j.psychres.2011.03.004, PMID: 21463903

[43] Li J Cai QC Dai Z Lin J Chen BC Liu SJ. Study on the insomnia among military medical university students. Chin J Sch Health. (2004) 25:19–20.

[44] Jayakody K Gunadasa S Hosker C. Exercise for anxiety disorders: systematic review. Br J Sports Med. (2014) 48:187–96. 23299048 10.1136/bjsports-2012-091287

[45] Kim S-H. The effects of physical leisure activities on stress level and solitude, life satisfaction of old woman in an asylum. Korean Soc Sport Sci. (2008) 17:367–74.

[46] Bai Y. The relationship among loneliness, social anxiety and mobile phone dependence in college students: a cross-lag analysis. J Clin Psychol. (2022) 30:64–7. doi: 10.16128/j.cnki.1005-3611.2022.01.013

[47] Wang Y Chen J Zhang X Lin X Sun Y Wang N. The relationship between perfectionism and social anxiety: a moderated mediation model. Int J Environ Res Public Health. (2022) 19:12934. doi: 10.3390/ijerph191912934, PMID: 36232234 PMC9566146

[48] Sonstroem RJ. Exercise and self-esteem. Exerc Sport Sci Rev. (1984) 12:123–56. doi: 10.1249/00003677-198401000-00007 6376132

[49] White K Kendrick T Yardley L. Change in self-esteem, self-efficacy and the mood dimensions of depression as potential mediators of the physical activity and depression relationship: exploring the temporal relation of change. Ment Health Phys Act. (2009) 2:44–52. doi: 10.1016/j.mhpa.2009.03.001

[50] Woo H-J. A study on the influence of mobile phone users’ self-traits on mobile phone addiction: focusing on self-esteem, self-efficacy, and self-control. Korean J Broadcast Telecom. (2007) 21:391–427.

[51] Wu W Guo Z Li S Tu F Wu X Ma X. The influence of parental autonomy support on cyberbullying victimization of high school students: a latent moderation analysis. Acta Psychol. (2022) 230:103739. doi: 10.1016/j.actpsy.2022.103739, PMID: 36088897

[52] Fergus S Zimmerman MA. Adolescent resilience: a framework for understanding healthy development in the face of risk. Annu Rev Public Health. (2005) 26:399–419. doi: 10.1146/annurev.publhealth.26.021304.144357 15760295

[53] Yang G Shangguan RL Ke YY Wang ST. The influence of acute aerobic exercise on craving degree for university students with mobile phone dependency: a randomized controlled trial. Int J Environ Res Public Health. (2022) 19:8983–91. doi: 10.3390/ijerph19158983, PMID: 35897357 PMC9331807

[54] Kaya MS. The use of dynamic cognitive Behavioural therapy (DCBT) in social anxiety disorder (SAD): a theoretical integration initiative. Med Lith. (2022) 58:1759. doi: 10.3390/medicina58121759, PMID: 36556961 PMC9781870

[55] Castillo I Molina-García J Estevan I Queralt A Álvarez O. Transformational teaching in physical education and Students’ leisure-time physical activity: the mediating role of learning climate, passion and self-determined motivation. Int J Environ Res Public Health. (2020) 17:4844. doi: 10.3390/ijerph17134844, PMID: 32635673 PMC7370029

